# 6.K. Workshop: Health Literacy in Health Care Settings in Europe: Practice experience, challenges and future needs

**DOI:** 10.1093/eurpub/ckac129.377

**Published:** 2022-10-25

**Authors:** Organised by: Fulda University of Applied Sciences (Germany), Chair persons: Katharina Rathmann (Germany)

## Abstract

Health care organizations (e.g., hospitals, nursing homes, facilities for people with disparities) are increasingly required to create health literate structures and processes, to train staff in such a way that they make it easier for their users to behave in a health literate manner, to deal competently with health information and to navigate competently through facilities and the entire health care system. This approach is known as the concept of organizational health literacy (OHL). Meanwhile, different standards (fields of action) of organizational health literacy and assessment tools to measure health literacy on an organizational level are available, which address, among other issues, health literacy as part of the quality management system, the development of health-related information by involving users, or to facilitate navigation within and to health care facilities and through the health care system. In order to assess and to strengthen OHL in health care settings, an increasing number of projects in Europe and internationally conduct needs assessments and interventions to implement tools to strengthen OHL at the patient-/client-, staff- and organizational-level. The assessment and implementation is accompanied by different requirements, challenges and needs at different levels (i.e. clients, staff, management).

Thus, the workshop emphasizes the following objectives:

1. to present findings from OHL projects in European countries (Austria, Belgium, Germany, the Netherlands, Switzerland).

2. to present and to discuss methodological issues (e.g., needs assessment),

3. to highlight practical experiences when implementing measures to strengthen OHL, and

4. to discuss beneficial factors, challenges and helpful approaches (e.g., participatory or co-creation setups) for the implementation of OHL.

A total of five experts on the topic of ‘health literacy in health care settings’ will present and discuss the implementation of health literacy in health care settings in Central European countries.

**Key messages:**

• The Workshop highlights a synthesis of challenges and future needs that are evident for organizations and users at different levels (management, staff, clients).

• Beneficial factors are a supporting management and leadership staff, participatory approaches, communication tools, training staff in health literacy skills as well as clients/patients.

